# Role of the DECAF Score in Predicting In-hospital Mortality in Acute Exacerbation of Chronic Obstructive Pulmonary Disease

**DOI:** 10.7759/cureus.4826

**Published:** 2019-06-04

**Authors:** Mubeen A Memon, Sheeba Faryal, Naveed Brohi, Besham Kumar

**Affiliations:** 1 Pulmonology, Civil Hospital, Jamshoro, PAK; 2 Intenal Medicine, Civil Hospital, Jamshoro, PAK; 3 Internal Medicine, Jinnah Postgraduate Medical Center, Karachi, PAK

**Keywords:** predictors of mortality, in-hospital mortality, copd, copd exacerbation, acute exacerbation of copd, decaf score

## Abstract

Introduction

Acute exacerbations of chronic obstructive pulmonary disease (AECOPD) can be fatal. In 2012, a comprehensive score was developed to predict the risk of in-hospital mortality in AECOPD called the dyspnoea, eosinopenia, consolidation, acidemia, and atrial fibrillation (DECAF) score. We conducted this study to assess the value of the DECAF score as a clinical prediction tool that claims to stratify all patients with AECOPD by risk accurately.

Methods

We conducted a prospective study of patients admitted to the intensive care unit (ICU) of the Department of Pulmonology in Civil Hospital, Jamshoro, from January 2016 to December 2018. Our inclusion criteria were that the patient must be age 35 years or older, have a primary clinical diagnosis of AECOPD, spirometry consistent with airflow obstruction, and have a smoking history of ≥10 cigarette pack per year. We excluded patients who had domiciliary ventilation, survival-limiting comorbidities (such as metastatic malignancy), and a primary reason for admission other than AECOPD. All sociodemographic data were collected at the time of admission, including age, gender, co-morbidities, housebound status, and number of previous AECOPD. Clinical data collected included plain chest x-ray, spirometry, electrocardiogram, arterial blood gases analysis, complete blood count, kidney function test, liver function test, and serum electrolytes. A DECAF score was applied to each patient. We noted in-hospital mortality and compared the characteristics of survivors and non-survivors. Data were analyzed using IBM SPSS for Windows, version 19.0 (IBM Corp, Armonk, NY).

Results

A total of 162 patients were included in the study. The mortality rate was 13% (n=21). More survivors had a DECAF score from zero to three than non-survivors. The difference in the number of survivors vs. non-survivors was statistically significant for DECAF scores zero and one. For DECAF scores four and five, there were more patients in the “non-survivors” group, and the differences were statistically significant. None of the patients scored six on DECAF.

Conclusion

Patients with a DECAF score of four or higher have a significant risk of mortality. DECAF is a simple tool that predicts mortality that incorporates routinely available indices to stratify AECOPD patients into mortality risk categories.

## Introduction

Chronic obstructive pulmonary disease (COPD) is among the more common noncommunicable diseases in the field of pulmonology. It is characterized by limited airflow and has a debilitating impact on both quality of life and life expectancy [1]. COPD is the third-leading cause of death [2]. COPD has a chronic, stable pattern aggravated by sudden, acute episodes of dyspnea and productive cough. These acute exacerbations of COPD (AECOPD) may be triggered by tobacco smoking or air pollution [2] and can be fatal [3]. AECOPD become more frequent as COPD progresses and have a high recurrence risk within eight weeks. The in-hospital mortality rate for AECOPD may range from 2.5% to 25%; readmission rates may be from 25% to 55% for those who survived, and 25% to 50% of these patients may die within one year [4]. The single most crucial parameter to determine the risk of mortality in patients experiencing AECOPD is the forced expiratory volume in one second (FEV1). Other risks include hypoxemia or hypercapnia upon arterial blood gas (ABG) analysis, short distance walked in a fixed time, the severity of functional dyspnea, and low body mass index [1].

There is extensive literature available on the prognostic indices for stable COPD, and instruments have been standardized, after thorough clinical investigations, for predicting the risk of mortality. One such score is the BODE index (i.e., body mass index, airflow obstruction, dyspnea, and exercise) [1]. However, the data on prognostic instruments in settings of AECOPD are not well-established. Steer et al. in 2010 studied this potential gap in the scientific data and noted that individual predictors of mortality were robust enough to be standardized for AECOPD [4]. Steer later developed a comprehensive score to predict the risk of in-hospital mortality in AECOPD called the DECAF score [3]. DECAF consists of five parameters: dyspnea (D), eosinopenia (E), consolidation (C), acidemia (A), and atrial fibrillation (F). DECAF is a simple tool that can be administered at the bedside using indices routinely available on admission. Administering DECAF in severely ill patients can help to predict mortality. Identified high-risk patients must be closely monitored with efficient and timely medical interventions to help reduce the overall mortality rate. We conducted this study to evaluate the DECAF score as a clinical prediction of mortality for patients with AECOPD.

## Materials and methods

We conducted this prospective study at the intensive care unit (ICU) of the Department of Pulmonology, Civil Hospital, Jamshoro, from January 2016 to December 2018. The study design was approved by the institutional review board. The study included patients aged 35 years or older who were admitted to the ICU and had a primary clinical diagnosis of AECOPD, spirometry consistent with airflow obstruction (FEV1/forced vital capacity <0.70), and a smoking history of ≥10 cigarette packs per year. Patients excluded from the study were those with domiciliary ventilation, survival-limiting comorbidity (e.g., metastatic malignancy), or those whose primary reason for admission was something other than AECOPD.

Sociodemographic data including age, gender, comorbidities, housebound status, and number of previous AECOPD were collected on admission. Clinical data were also collected on admission and consisted of plain chest x-ray, spirometry, electrocardiogram, ABG analysis, complete blood count, kidney function test, liver function test, and serum electrolytes. A DECAF Score [1] was calculated for each patient according to Table 1.

**Table 1:** DECAF score calculation DECAF: dyspnea, eosinopenia, consolidation, acidemia, and atrial fibrillation; eMRCD, extended Medical Research Council dyspnea score [2].

Variables (DECAF)		Score
Dyspnea		
	eMRCD 5a (too breathless to leave the house unassisted but independent in washing and/or dressing)	1
	eMRCD 5b (too breathless to leave the house unassisted and requires help with washing and dressing)	2
Eosinopenia (eosinophils <0.05×10^9^/L)		1
Consolidation		1
Moderate or severe acidemia (pH <7.3)		1
Atrial fibrillation (including history of paroxysmal atrial fibrillation)		1
Maximum DECAF score		6

Data were analyzed using IBM SPSS for Windows, Version 19.0 (IBM Corp, Armonk, NY). We compared survivors to non-survivors. Mean and standard deviation was calculated for continuous variables. The frequency of the sociodemographic and clinical characteristics of both study groups were compared; chi-square was applied. P<0.05 was considered significant. The frequency of each score for survivors and non-survivors were calculated and compared.

## Results

A total of 162 patients were included in the study. The mean age of these patients was 69 ± 5 years. There were 93 (57.4%) men and 69 (42.5%) women. There were 65 (40.1%) participants who were housebound due to the severity of their disease. The mean duration of hospital stay was 4 ± 3 days. In total, 141 patients (87.0%) survived the AECOPD, were successfully managed, and discharged to home. Twenty-one patients (13.0%) died in the hospital.

We compared the admission characteristics of survivors and non-survivors. We noted that men were more common in both groups. Non-survivors were more likely to be housebound and on long-term oxygen therapy. Non-survivors were also older and had lower mean FEV1 scores. The differences were statistically significant as shown in Table 2.

**Table 2:** Comparison of the characteristics of survivors and non-survivors at the time of admission SD, standard deviation; AECOPD, acute exacerbations of chronic obstructive pulmonary disease; FEV1, forced expiratory volume in one second; eMRCD, extended Medical Research Council dyspnea score.

Patient characteristics at the time of admission	Survivors (n=141)	Non-survivors (n=21)	P-value
Gender (male/female)	82 (58.2%) / 59 (41.8%)	11 (52.3%) / 10 (47.6%)	
Housebound	46 (32.6%)	19 (90.47%)	0.000
Age in years (mean ± SD)	66.5 ± 7.23	71.3 ± 8.48	0.0006
Number of previous AECOPD (mean ± SD)	3.0 ± 0.71	3.2 ± 1.01	0.26
FEV_1 _(mean ± SD)	43 ± 12.71	39 ± 13.11	0.04
Long-term oxygen therapy (%)	19 (13.47%)	9 (42.8%)	0.0008
eMRCD, median (range)	4 (3-5b)	5 (5a-5b)	
Co-morbidity status			
Stroke	13 (9.21%)	2 (9.52%)	0.96
Ischemic heart disease	50 (35.46%)	7 (33.33%)	0.85
Heart	60 (42.55%)	9 (42.85%)	0.98
Diabetes	24 (17.02%)	3 (14.28%)	0.75
Atrial fibrillation	15 (10.6%)	9 (42.85%)	0.0001
Renal comorbidities	18 (12.76%)	4 (19.04%)	0.43

A comparison of the clinical and radiological characteristics of survivors and non-survivors is presented in Table 3. In the survivors group, patients were more frequently seen to have purulent sputum and were less likely to have ineffective cough. Acute confusion and consolidation on chest x-ray was more commonly seen in non-survivors. We witnessed that non-survivors had a higher mean respiratory rate and mean partial pressure of carbon dioxide. The differences were statistically significant as shown in Table 3.

**Table 3:** Comparison of the clinical and radiological characteristics of survivors and non-survivors at the time of admission *Mean and standard deviation calculated. paO2, partial pressure of oxygen; paCO2, partial pressure of carbon dioxide; HCO3, hydrogen bicarbonate.

Clinical and radiological characteristics	Survivors (n=141)	Non-survivors (n=21)	P-value
Purulent sputum	93 (65.9%)	7 (33.3%)	0.006
Ineffective cough	18 (12.7%)	9 (42.8%)	0.002
Acute confusion	15 (10.6%)	10 (47.6%)	0.00015
Heart rate/min*	101.3 ± 20.2	102.1 ± 21.6	0.61
Systolic blood pressure*	141.5 ± 27.21	137.7 ± 26.12	0.55
Diastolic blood pressure*	78.3 ± 16.8	76.4 ± 17.8	0.63
Respiratory rate/min*	24.5 ± 5.24	28.1 ± 6.2	0.0047
Temperature (Celsius)*	37.0 ± 0.56	36.89 ± 0.51	0.40
Body mass index (kg/m^2^)*	24.23 ± 5.2	22.81 ± 6.1	0.25
Radiological consolidation	49 (34.7%)	16 (76.2%)	0.0005
Arterial blood gases analysis			
pH*	7.42 ± 0.09	7.31 ± 0.06	< 0.0001
paO_2 _(mm Hg)*	66.23 ± 10.2	64.12 ± 11.12	0.38
paCO_2 _(mm Hg)*	42.52 ± 7.1	49.81 ± 12.46	0.0001
HCO_3 _(mm Hg)*	28.23 ± 5.6	27.81 ± 6.1	0.75
Oxygen saturation (%)*	92.12 ± 5.2	92.23 ± 5.3	0.92

We calculated DECAF scores for all patients, and a DECAF score of three was most common (n=44; 27.2%). The rate of mortality was 13% (n=21). On DECAF score zero, 2.9% of patients died, and 97.1% survived (p=0.04). On DECAF score one, 2.6% of patients died and 97.3% survived (p=0.03). On DECAF scores two and three, 9.7% and 15.9% of patients died, respectively, and 90.3% and 84.1% survived, respectively. The difference was not statistically significant. On DECAF scores four and five, 60% and 75% patients died, respectively, and 40% and 25% survived, respectively (p<0.00001; p=0.0001). No patients had a DECAF score of six (Table 4).

**Table 4:** Comparison of the DECAF scores of survivors and non-survivors DECAF: dyspnea, eosinopenia, consolidation, acidemia, and atrial fibrillation

DECAF Score	Total study sample (N=162)	Non-survivors (N=21)	Survivors (N=141)	P value
0	35 (21.6%)	1 (2.9%)	34 (97.1%)	0.04
1	38 (23.4%)	1 (2.6%)	37 (97.3%)	0.03
2	31 (19.1%)	3 (9.7%)	28 (90.3%)	0.54
3	44 (27.2%)	7 (15.9%)	37 (84.1%)	0.49
4	10 (6.2%)	6 (60.0%)	4 (40.0%)	<0.00001
5	4 (2.5%)	3 (75.0%)	1 (25.0%)	0.0001
6	0 (0%)	0 (0%)	0 (0%)	

## Discussion

COPD is among the more common and debilitating non-communicable lung diseases. The disease is even more severe in middle- and low-income countries due to respiratory exposures associated with poverty and lack of hygiene, restricted access to healthcare, inadequate knowledge about the health hazards of smoking, and difficulty seeking medical care due to various reasons including lack of funds and health insurance [5]. In such settings, it becomes necessary to customize investigation and treatment modalities to make them cheaper, easily accessible, and readily administrable. The DECAF score system is a predictor of in-hospital mortality associated with AECOPD which uses clinical, serological, and radiological parameters. According to this study, DECAF scores of 0-1 are strong predictors of survival, and DECAF scores of 4-6 are strong predictors of mortality.

The rate of mortality in this study was 13% which was comparable to other studies which have reported a mortality rate of 10.4% [3], 7.7% [6], and 12.5% [7]. In a comprehensive analysis of patients with AECOPD, the highest mortality was seen in patients with DECAF scores of 3-5 (92%). The in-hospital mortality rate increased with every point increase in the DECAF score [8]. In another study with an 18% mortality rate in patients with AECOPD, patients who died scored higher on both DECAF and the BAP-65 metric (B: elevated blood urea nitrogen, A: altered mental status, P: pulse >109, 65: age >65 years). The sensitivity of the DECAF score and BAP-65 score for predicting mortality was 100%, and specificity was 34.1% and 63.4%, respectively [9]. However, a detailed comparison of the two scales suggests that DECAF is superior to BAP-65 [10]. In another study, most of the patients with 30-day mortality scored four on DECAF; a higher DECAF score was significantly associated with the 30-day mortality score [11]. The DECAF score has also been used to gauge the length of hospital stay in patients with AECOPD; as the DECAF score increased, the length of hospital stay also increased [12-13].

The DECAF score is a clinical, serological, and radiological scale which can be effectively utilized in predicting mortality risk in patients with acute exacerbation of COPD. The widespread use of the DECAF score in pulmonology can help identify high-risk patients at the level of emergency room triage. Urgent medical interventions and intensive care for the patients identified through DECAF can help reduce AECOPD mortality.

Our study was limited in that we did not assess the relationship between the DECAF score and length of hospital stay. Additional future DECAF studies with robust methodologies and larger samples including multiple tertiary care centers across Pakistan are warranted.

## Conclusions

DECAF is a simple tool that predicts mortality that incorporates routinely available indices. It effectively stratifies COPD patients admitted with acute exacerbations into mortality risk categories. DECAF scores of zero to one are strong predictors for survival, and DECAF scores of four to six are strong predictors of mortality. The DECAF system can facilitate the efficient management of patients with AECOPD.
